# Supplementary material for: Inflammatory factors and risk of lung adenocarcinoma: a Mendelian randomization study mediated by blood metabolites
Source: Front Endocrinol (Lausanne). 2024 Aug 27;15:1446863. doi: 10.3389/fendo.2024.1446863 (PMC11384989; doi:10.3389/fendo.2024.1446863)
Supplement: Supplementary file 1 [file DataSheet1.zip › supplementary files/S2/GCST90200146/forest.pdf]

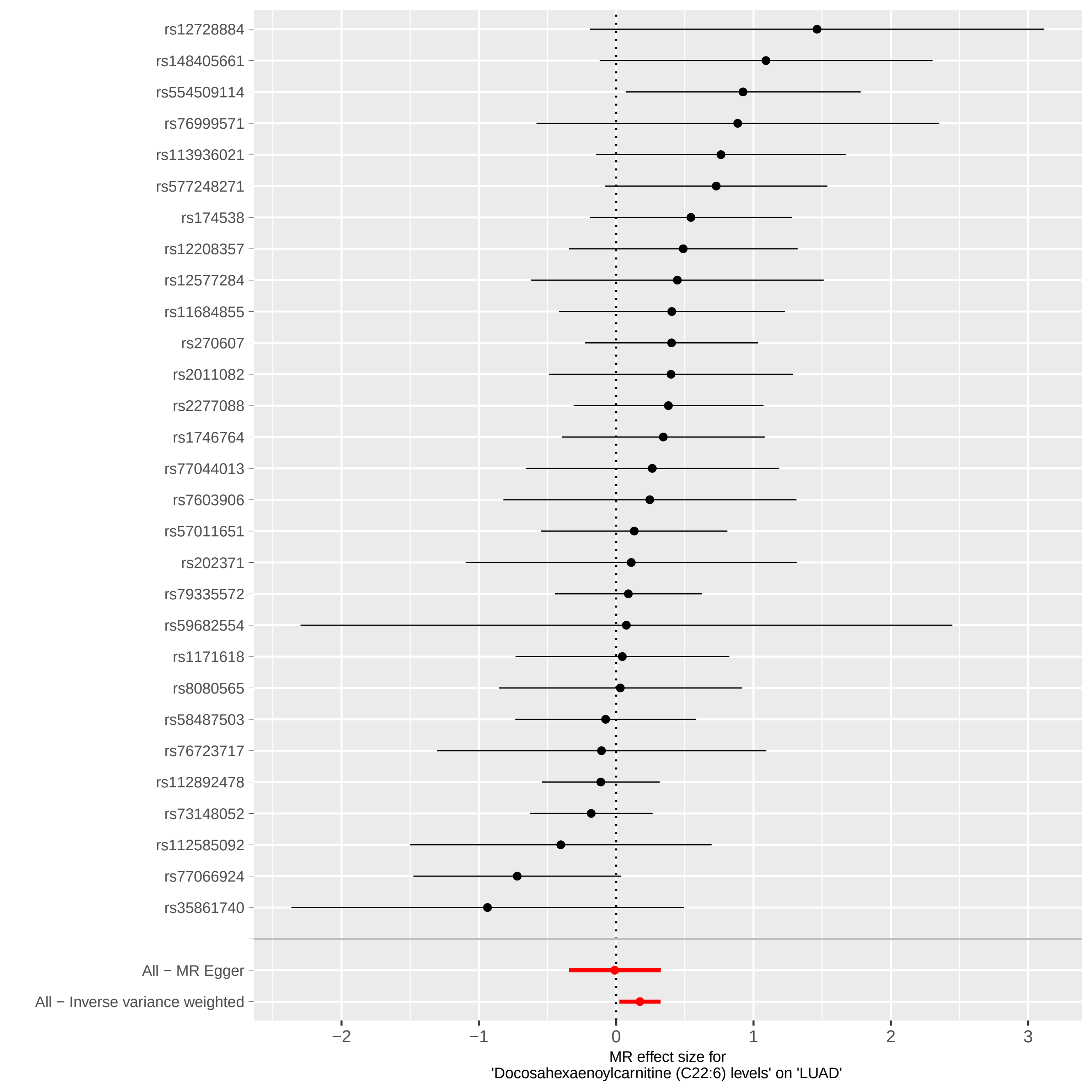
rs12728884
rs148405661
rs554509114
rs76999571
rs113936021
rs577248271
rs174538
rs12208357
rs12577284
rs11684855
rs270607
rs2011082
rs2277088
rs1746764
rs77044013
rs7603906
rs57011651
rs202371
rs79335572
rs59682554
rs1171618
rs8080565
rs58487503
rs76723717
rs112892478
rs73148052
rs112585092
rs77066924
rs35861740
All − MR Egger
All − Inverse variance weighted
−2
−1
0
1
2
3
MR effect size for
'Docosahexaenoylcarnitine (C22:6) levels' on 'LUAD'
